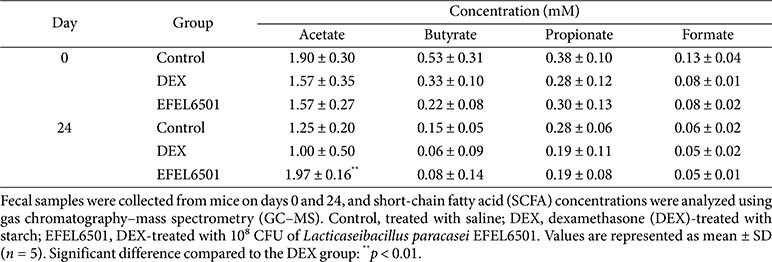# Gut Microbiota Modulation by *Lacticaseibacillus paracasei* EFEL6501 Ameliorates Muscle Atrophy

**DOI:** 10.4014/jmb.2508.08024

**Published:** 2025-11-26

**Authors:** Geum Na Pyeon, Hyunbin Seong, Jin Seok Moon, Nam Soo Han

**Affiliations:** 1 Brain Korea21 Center for Smart GreenBio Convergence and Sustainable Regional Development, Division of Animal, Horticultural, and Food Sciences, Chungbuk National University, Cheongju 28644, Republic of Korea; 2 Ildong Pharmaceutical Co., Ltd., Seoul 06752, Republic of Korea" to "Ildong Bioscience, Pyeongtaek-si, Gyeonggi-do, 17957, Republic of Korea

**Keywords:** Muscle atrophy, probiotic, gut microbiota, short-chain fatty acid, gut-muscle axis

## Abstract

Probiotics play a crucial role in promoting host health by modulating the composition of the gut microbiota through the production of their own bioactive metabolites. The aim of the study was to investigate the anti-atrophic effects of *Lacticaseibacillus paracasei* EFEL6501 (EFEL6501) in dexamethasone (DEX)-treated C2C12 myotubes and a mouse model. *In vitro* experiments demonstrated that specific bioactive metabolites present in the cell culture supernatant (CS) and lysate supernatant (LS) of EFEL6501 alleviated muscle degradation and restored muscle protein synthesis in DEX-induced C2C12 myotubes. Similarly, EFEL6501 supplementation in mice significantly enhanced muscle thickness (6.09 mm), grip strength (117.87 g), and the cross-sectional area (CSA) (34.11 μm^2^) of the gastrocnemius muscle, compared to the DEX group (5.70 mm, 106.87 g and 29.79 μm^2^, respectively), by suppressing protein degradation pathways and improving muscle differentiation. Furthermore, EFEL6501 positively modulated the gut microbiota composition by increasing the abundance of beneficial bacteria, including *Lactobacillus reuteri* (7.19%), *Bifidobacterium choerinum* (25.66%), *Bacteroides uniformis* (0.29%), *Allobaculum* (0.63%), and *Faecalibaculum* (18.00%) compared to the DEX group (3.44%, 0.75%, 0.14%, -0.63%, and 8.53%, respectively), while also elevating acetate concentrations from 1.57 ± 0.27 mM to 1.97 ± 0.16 mM. Taken together, EFEL6501 may serve as a potential functional probiotic for preventing muscle atrophy by regulating muscle metabolism and gut microbiota composition.

## Introduction

Skeletal muscle constitutes approximately 40% of total body weight and plays a crucial role in movement and energy metabolism by contributing 30–50% to whole-body protein turnover [1]. Muscle mass and strength depend on the balance between muscle protein synthesis and degradation [2]. However, muscle protein imbalance caused by factors such as nutritional status, hormonal balance, physical activity, and disease can accelerate protein degradation, leading to muscle loss. This process is further exacerbated by aging, as skeletal muscle undergoes fiber atrophy and fiber loss, ultimately resulting in a progressive decline in muscle mass [3, 4]. Consistent with this, evidence suggests that muscle mass declines by 3–8% annually after the age of 30, with the rate of loss accelerating beyond the age of 60 [5-7]. This deterioration contributes to functional impairments and increased vulnerability to muscle-related disorders.

Skeletal muscle atrophy is classified into primary atrophy, which results from congenital or genetic disorders, and secondary atrophy, which is caused by acquired factors such as pathological or physiological conditions [8]. Age-related muscle atrophy, also known as sarcopenia, is considered a form of secondary atrophy resulting from physiological aging [9]. Muscle fibers are generally categorized into slow oxidative (type I), fast oxidative (type IIa), and fast glycolytic (type IIb) types. Aging mainly induces the atrophy of type II fibers, leading to a characteristic shift from type II to type I fibers [10, 11]. The lack of normal muscle contraction and anabolic stimulation activates muscle atrophy signaling, leading to protein degradation and cell apoptosis [12]. Ultimately, muscle atrophy significantly impairs the quality of life and physical disability in the elderly by increasing the risk of falls and fractures, which can lead to reduced mobility, prolonged hospitalization [13].

To date, no pharmacological treatments have been approved for the prevention or treatment of sarcopenia, and regular exercise and dietary management are recommended as primary strategies [14]. In contrast, recent reports have increasingly suggested a strong association between the gut microbiota and sarcopenia in the elderly [15]. The gut microbiome plays a crucial role in modulating the immune response [16], enhancing the intestinal barrier, regulating intestinal permeability [17], and blocking pathogen invasion [18], which may suggest its significant impact on muscle health. Meanwhile, the imbalance in gut structure and microbiota caused by aging is closely associated with a decrease in muscle mass and strength [19]. This implies a reduction in the microbial composition typically found in healthy individuals due to aging, along with a decline in microbial metabolites that can physiologically impact muscle health [20, 21]. The results of these findings support the potential of probiotic supplementation in improving muscle strength and physical function, which are compromised by aging or immobility. Several studies demonstrated that *Limosilactobacillus gasseri* IM13-fermented whey protein protects against dexamethasone (DEX)-induced muscle atrophy by enhancing myogenesis and muscle protein synthesis while inhibiting protein degradation via the insulin-growth factor-1 (IGF-1)/ phosphatidylinositol 3-kinase (PI3K)/serine/threonine kinase 1 (AKT) pathway [22]. Additionally, *Lactiplantibacillus plantarum* KM-2-derived postbiotic KL-Biome mitigates DEX-induced muscle atrophy by modulating skeletal muscle degradation pathways and regulating gut microbiota composition [23]. Furthermore, *Lacticaseibacillus casei* Shirota alleviated age-related sarcopenia by enhancing muscle strength, mitigating mitochondrial dysfunction, and regulating the gut microbiota to promote short-chain fatty acid (SCFA) production [24]. Nevertheless, most studies have predominantly focused on the direct effects of probiotics on muscle homeostasis, whereas investigations into gut microbiota composition and gut-derived metabolites are still limited. Accordingly, further research is required to elucidate the role of probiotic strains in modulating gut microbiota composition and metabolite production, and their subsequent effects on muscle physiology.

The species *Lacticaseibacillus paracasei* is a Gram-positive, homofermentative lactic acid bacterium that is widely utilized in dairy product fermentation and probiotic culture applications. This strain is commonly found in various environments, including the human intestinal tract, oral cavity, and dairy products [25]. *L. paracasei* produces bioactive compounds associated with health benefits, such as extracellular polysaccharides (EPS) and lipoteichoic acid (LTA), which have been reported to exhibit strong anti-inflammatory [26, 27], anticancer [28], and antioxidant effects [29]. Based on these characteristics, several studies have demonstrated their therapeutic effects for various health disorders. For instance, *L. paracasei* Jlus66 exerts protective effects against colitis by modulating gut microbiota composition, promoting anti-inflammatory immune responses, and enhancing intestinal barrier function [30]. Furthermore, *L. paracasei* sh2020 enhances the efficacy of anti-PD-1 immunotherapy and boosts antitumor immune responses by modulating gut microbiota and gut barrier function [31]. Although numerous studies have demonstrated the health-promoting effects of *L. paracasei*, limited attention has been given to its potential role in modulating the gut microbiome with implications for muscle health. In this context, the present study aims to address this gap by investigating whether *L. paracasei* EFEL6501 can modulate gut microbiota composition and gut-derived metabolites in a DEX-induced muscle atrophy model, and how these changes are linked to improvements in muscle mass, strength, and molecular markers of muscle homeostasis.

Therefore, this study aimed to investigate the anti-atrophic effects of *L. paracasei* EFEL6501 (EFEL6501) in DEX-treated C2C12 myotubes and a mouse model. *In vitro* experiments using C2C12 myotubes evaluated the expression of genes related to myogenesis and muscle atrophy. Additionally, a DEX-treated mouse model was used to assess muscle function and morphology through grip strength measurements and Sirius Red staining. To elucidate underlying mechanisms, mRNA levels of muscle-related markers and concentrations of myostatin and IGF-1 were measured via real-time quantitative polymerase chain reaction (qPCR) and enzyme-linked immunosorbent assay (ELISA). Additionally, gut microbiota composition and bioactive metabolites were analyzed using metagenomic sequencing and gas chromatography–mass spectrometry (GC–MS), respectively.

## Materials and Methods

### Preparation of *Lacticaseibacillus paracasei* EFEL6501

*L. paracasei* EFEL6501 (EFEL6501, KCTC 14859BP) was isolated from fermented food sources. For cell-based experiments, EFEL6501 was inoculated at 2% into de Man-Rogosa-Sharpe (MRS; BD Difco, USA) broth and cultured at 37°C for 18 h. The cell culture supernatant (CS) was obtained by removing the cell pellet via centrifugation, followed by filtration of the supernatant through a 4.5-μm pore-size filter. Additionally, the cell lysate supernatant (LS) was prepared by autoclaving the bacterial cell pellet at 121°C for 15 min, followed by centrifugation and filtration using a 4.5-μm pore-size filter. For animal experiments, EFEL6501 cells were harvested by centrifugation, and resuspended in phosphate-buffered saline (PBS) buffer. The freeze-dried EFEL6501 powders, prepared using starch as a cryoprotectant, were resuspended in PBS and administered to animals daily.

### Cultivation and Differentiation of C2C12 Myoblast Cells

C2C12 myoblast cell line (American Type Culture Collection, Manassas (ATCC), USA) was cultured in Dulbecco’s modified Eagle’s Medium (DMEM; GenDEPOT, USA) containing 10% fetal bovine serum (FBS; ATCC, USA) and 1% penicillin/streptomycin (P/S, GenDEPOT, USA) at 37°C and 5% CO_2_. To induce C2C12 cell differentiation into myotubes, the differentiation medium was replaced with DMEM containing 2% horse serum (Thermo Fisher Scientific, USA) and 1% P/S. The differentiation medium was replaced daily. A glucocorticoid-induced muscle atrophy cell model was established by treating cells with 5 μM dexamethasone (DEX; Sigma-Merck, USA) for 24 h.

### Animal Experiment

Animal experiments were approved by the Institutional Animal Care and Use Committee (IACUC) at Gyeongsang National University, Korea (approval No. GUN-241126-M00227). Six-week-old male C57BL/6 mice were obtained from Samtako Bio Korea (Republic of Korea) and housed under conventional conditions (23 ± 2°C, 60 ± 5% humidity, and a 12-h light/dark cycle). After a week of acclimatization, the mice were randomly allocated into three groups (*n* = 8/group), including control group, DEX-treated group (DEX), and EFEL6501-treated group (EFEL6501). From day 0 to day 24, the control group received an equal volume of saline, the DEX group was administered the same amount of starch used as a cryoprotectant, and the EFEL6501 group was orally gavaged once daily with 10^8^ colony-forming units (CFU) of EFEL6501 suspended in 300 μl of saline. From day 15 to day 24, DEX (25 mg/kg) was intraperitoneally injected once daily into both the DEX and EFEL6501 groups. During the treatment period, grip strength was measured at the same time on days 0, 20, and 24. On day 25, the mice were sacrificed, and skeletal muscle tissues (gastrocnemius, plantaris, and soleus) were excised, weighed, and preserved at −80°C for subsequent analysis.

### Total RNA Preparation and Reverse Transcription Quantitative PCR

Total RNA was extracted from C2C12 myotube cells and mouse gastrocnemius muscle using Trizol (Invitrogen, USA) according to the manufacturer’s protocol. Subsequently, RNA was reverse transcribed into cDNA using the PrimeScript First Strand cDNA Synthesis Kit (Takara, Japan). Real-time qPCR was performed using AccuPower 2X GreenStar qPCR Master Mix in an Exicycler 96 Real-Time Quantitative Thermal Block (Bioneer, Republic of Korea), and the primer sequences used for real-time qPCR are listed in Table 1. RT-qPCR analysis was performed in triplicates for each sample. The PCR conditions were as follows: 95°C for 5 min, followed by 40 cycles at 95°C for 15 sec, 60°C for 30 sec, and a final extension at 60°C for 30 sec. Glyceraldehyde-3-phosphate dehydrogenase (GAPDH) was used as a housekeeping gene to normalize the expression levels of the target gene, and relative mRNA expression was determined using the delta-delta Ct (ΔΔCt) method.

### Serum Biochemical Analysis

After a 12 h fasting period, blood samples were collected from the abdominal aorta, and the mice were subsequently sacrificed. Serum was separated by centrifuging the blood at 1,100 ×*g* for 20 min at 4°C and stored at -80°C. The concentration of serum myostatin was quantified using the GDF-8/Myostatin DuoSet ELISA kit (R&D Systems, USA). Serum insulin-like growth factor-1 (IGF-1) was determined using the IGF-I/IGF-1 DuoSet ELISA kit (R&D Systems) according to the manufacturers’ protocols. All experiments were performed in triplicate, and absorbance was recorded at 450 nm using a microplate reader (Agilent, USA).

### Evaluation of Muscle Performance

Muscle performance was assessed by measuring grip strength and muscle thickness. Forelimb grip strength was evaluated using a grip strength meter (JD-A-22, Jeung Do B&P, Republic of Korea), and gastrocnemius muscle thickness was measured using calipers (A&D, Republic of Korea). Measurements were conducted at the same time on days 0, 20, and 24. All data were obtained in triplicate, and the mean value was determined for each trial.

### Sirius Red Staining

Sirius Red staining was performed to observe the cross-sectional area of muscle fibers in DEX-induced muscle atrophy [32, 33]. After sacrifice, the right gastrocnemius muscle was excised and immediately fixed in 10% neutral buffered formalin. The fixed tissues were embedded in paraffin, deparaffinized, and stained with picro-sirius red (Sigma-Merck) followed by washing with distilled water and 1% acetic acid. The tissues were then dehydrated using ethanol, treated with xylene for clearing, and mounted onto slides. Muscle fiber cross-sectional area (CSA) was analyzed with ZEN 3.1 (Carl Zeiss Microscopy GmbH, Germany), ensuring that at least 200 fibers were counted per sample.

### Microbiome Analysis of Mouse Feces

Fecal samples from each mouse were collected on days 0 and 24, suspended in PBS buffer, and centrifuged at 11,000 ×*g* for 1 min. Bacterial genomic DNA (gDNA) was extracted using the Genomic DNA Prep Kit (SolGent, Republic of Korea) following the protocol provided by the manufacturer. The microbial communities in mouse fecal samples were analyzed using the Illumina MiSeq platform (Illumina, USA) based on tag-encoded 16S ribosomal RNA (16S rRNA) gene sequencing. For 16S rRNA gene amplification, the primer pair 341-F (5'-CCTACGGGNGGCWGCAG-3') and 785-R (5'-GACTACHVGGGTATCTAATCC-3') was used [34]. Raw sequences were trimmed using Cutadapt and subsequently analyzed through the Deblur pipeline in quantitative insights into microbial ecology (QIIME) [35, 36]. Taxonomic classification was performed using a Naïve Bayes classifier, incorporating weighting based on the SILVA 132 database to improve accuracy. Alpha diversity indices obtained from the QIIME 2 pipeline were visualized using the ggplot2 package in R.

### Analysis of Short-Chain Fatty Acids in Mouse Feces

Fecal samples from muscle atrophy-induced mice were collected on days 0 and 24 for metabolite analysis using GC-MS. Fecal samples were suspended in PBS buffer and centrifuged at 11,000 ×*g* for 1 min to obtain the supernatant. A butanol-based solvent containing 100 μM 4-methylvaleric acid was used to extract SCFAs from the supernatant. To acidify the sample, 200 μl of fecal supernatant was combined with 20 μl of 20% phosphoric acid, followed by the addition of 240 μl of butanol. Following centrifugation at 16,100 ×*g* for 10 min, the upper phase was isolated, and 40 mg of sodium sulfate was added to eliminate residual water. The mixture was then vortexed and subjected to an additional centrifugation at 16,100 ×*g* for 5 min. The final supernatant was analyzed using gas chromatography with a DB-WAX column (30 m × 0.25 mm × 0.25 μm, Agilent), employing helium as the carrier gas at a flow rate of 1.5 ml/min. Data acquisition was performed using MassHunter Qualitative Analysis 10.0 (Agilent), and peak quantification was conducted by comparing spectra with the NIST ver. 2011b library.

### Statistical Analysis

All experiments were performed in triplicate as described, and the results are expressed as the mean ± standard deviation (SD). Statistical analyses were conducted using IBM SPSS software version 27 (IBM Corp., USA). Independent t-tests were performed after verifying that the assumptions of normality and homogeneity of variance were satisfied. Pairwise comparisons among the three groups (control vs. DEX, DEX vs. EFEL6501, and control vs. EFEL6501) were performed using independent T-tests. Significant differences are denoted by distinct letters or symbols on the error bars. A *p*-value of less than 0.05 was considered statistically significant. Figures were generated using GraphPad Prism 8.0 (GraphPad Software, USA).

## Results

### EFEL6501 Suppresses the Muscle Atrophic Factors in DEX-Induced C2C12 Cells

**Fig. 1:**
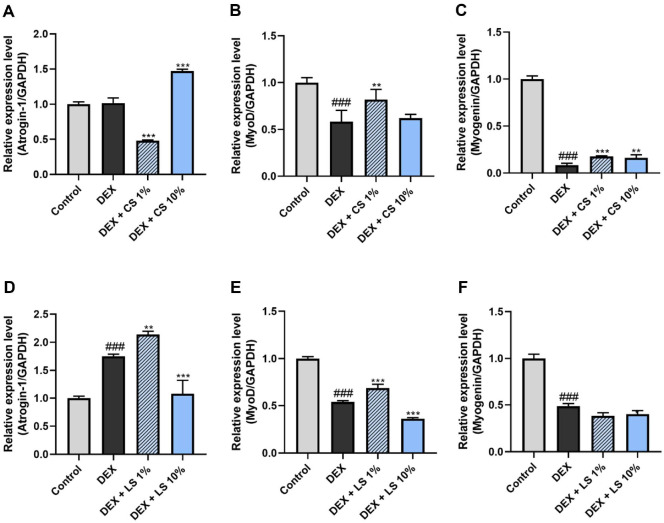
*Lacticaseibacillus paracasei* EFEL6501 promotes the expression of myogenesis-related genes and attenuates the expression of muscle-specific ubiquitin ligase in dexamethasone-induced C2C12 cells. Relative mRNA expression levels of (**A, D**) muscle atrophy F-box protein-1 (atrogin-1), (**B, E**) myogenic differentiation 1 (MyoD), and (**C, F**) myogenin. C2C12 myoblasts were induced to differentiate into myotubes and subsequently exposed to 5 μM dexamethasone (DEX). Cell culture supernatant (CS) and cell lysate supernatant (LS) of *Lacticaseibacillus paracasei* EFEL6501 (EFEL6501) were treated at concentrations of 1% and 10%. Values are represented as mean ± SD (*n* = 3). **p < 0.01, ***p < 0.001 vs. DEX group; ^###^*p* < 0.001 vs. control group.

To evaluate the anti-atrophic effects of the EFEL6501 strain under *in vitro* conditions, real-time qPCR was used to analyze the expression levels of myogenesis- and muscle degradation-related genes in DEX-induced C2C12 myotubes (Fig. 1). C2C12 myotubes were treated with either the CS or LS of EFEL6501 at final concentrations of 1% or 10%, respectively. MyoD and myogenin are known as myogenic regulatory factors, playing a crucial role in regulating myogenic differentiation [37, 38]. Muscle atrophy F-box (atrogin-1) and muscle-specific ring finger protein 1 (MuRF1) are key E3 ubiquitin ligases that regulate ubiquitin-mediated proteolysis in skeletal muscle [39]. The expression of MyoD and myogenin was reduced by DEX treatment, while the expression of Atrogin-1 was simultaneously increased. Conversely, as shown in Fig. 1A and 1D, treatment with 1% CS and 10% LS of EFEL6501 significantly reduced atrogin-1 expression (*p* < 0.001), thereby inhibiting muscle degradation. Moreover, treatment with 1% CS (*p* < 0.01) and LS (*p* < 0.001) significantly increased MyoD expression compared with the DEX group (Fig. 1B and 1E). In conclusion, EFEL6501 ameliorates DEX-induced muscle atrophy in C2C12 myotubes by promoting myogenic differentiation and inhibiting muscle protein degradation pathways. To validate these protective effects under physiological conditions, *in vivo* experiments were subsequently performed.

### EFEL6501 Restores Muscle Strength and Muscle Thickness Reduced by DEX-Induced Muscle Atrophy in Mice

**Fig. 2:**
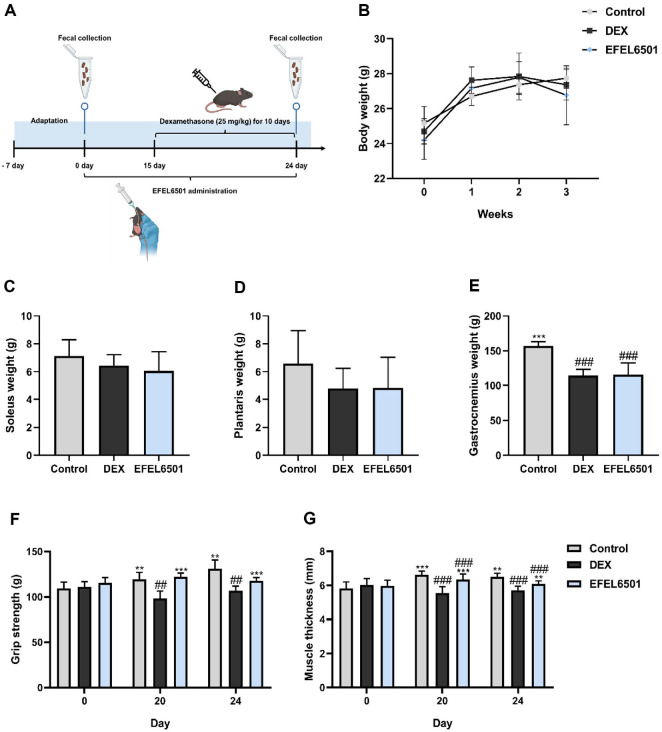
*Lacticaseibacillus paracasei* EFEL6501 ameliorates dexamethasone-induced muscle atrophy in mice. (**A**) Experimental scheme, (**B**) body weight (g), (**C**) soleus muscle weight (mg), (**D**) plantaris muscle weight (mg), (**E**) gastrocnemius muscle weight (mg), (**F**) grip strength (g), and (**G**) muscle thickness (mm). Six-week-old C57BL/6 mice were administered phosphate-buffered saline (PBS), starch, or *Lacticaseibacillus paracasei* EFEL6501 (EFEL6501) (10^8^ CFU/mouse/ day) daily for 24 days, followed by intraperitoneal injection of dexamethasone (DEX) once daily for 10 days after two weeks of probiotic administration. After 24 days, muscle tissues were collected following the sacrifice of the mice. Values are represented as mean ± SD (*n* = 5). **p < 0.01, ***p < 0.001 vs. DEX group; ^##^*p* < 0.01, ^###^*p* < 0.001 vs. control group. Control, treated with saline; DEX, DEX-treated with starch; EFEL6501, DEX-treated with 10^8^ CFU of *Lacticaseibacillus paracasei* EFEL6501.

To establish a DEX-induced muscle atrophy mouse model, a DEX solution (20 mg/kg) was intraperitoneally injected daily for 10 days, and EFEL6501 strain was orally administered to mice once daily for 24 days (Fig. 2A). Subsequently, 24 days after the induction of muscle atrophy, the effects of EFEL6501 were evaluated through measurements of body weight and muscle mass. As shown in Fig. 2B-2D, although body weight, soleus and plantaris weight appeared reduced in both the DEX and EFEL6501 groups compared with the control group, no significant differences were observed across the three groups (*p* > 0.05). In case of the gastrocnemius muscle weight, it was significantly decreased in both the DEX and EFEL6501 groups compared with the control group (*p* < 0.001), but no significant difference was found between the DEX and EFEL6501 groups (Fig. 2E). Meanwhile, treatment with EFEL6501 significantly restored forelimb grip strength at days 20 and 24 compared with the DEX group (*p* < 0.001) (Fig. 2F). Treatment with EFEL6501 significantly preserved muscle thickness on days 20 and 24 compared with the DEX group (*p* < 0.001 and *p* < 0.01, respectively), indicating attenuation of DEX-induced muscle loss (Fig. 2G). These findings suggest that EFEL6501 mitigates DEX-induced muscle atrophy by preserving muscle function, as evidenced by improved grip strength and reduced muscle thickness loss.

### EFEL6501 Exerts an Effect on the Expression of Genes Involved in Muscle Degradation and Differentiation in Muscle Tissue

**Fig. 3:**
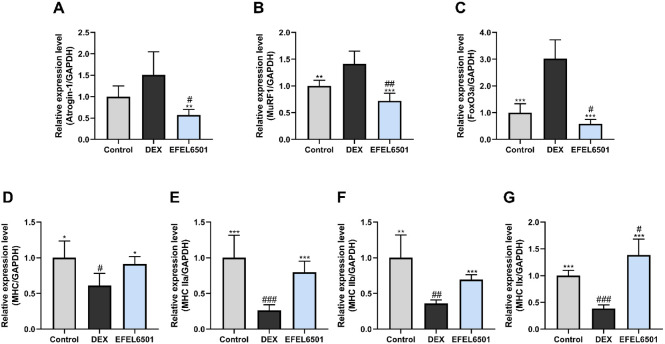
*Lacticaseibacillus paracasei* EFEL6501 downregulates muscle degradation genes and upregulates muscle differentiation genes in muscle tissue. The mRNA expression levels of (**A**) muscle atrophy F-box protein-1 (atrogin-1), (**B**) muscle RING-finger protein-1 (MuRF1), (**C**) forkhead box O3a (foxO3a), (**D**) myosin heavy chain (MHC), (**E**) MHC IIa, (**F**) MHC IIb, and (**G**) MHC IIx are shown. Values are represented as mean ± SD (*n* = 5). *p < 0.05, **p < 0.01, ***p < 0.001 vs. DEX group; ^#^*p* < 0.05, ^##^*p* < 0.01, ^###^*p* < 0.001 vs. control group. Control, treated with saline; DEX, dexamethasone (DEX)-treated with starch; EFEL6501, DEX-treated with 10^8^ CFU of *Lacticaseibacillus paracasei* EFEL6501.

The myosin heavy chain (MHC), a key contractile protein in the sarcomeric thick filament, is essential for sustaining muscle contraction [40]. Moreover, the forkhead box O (FOXO) signaling pathway is a critical regulator of skeletal muscle atrophy, primarily modulating E3 ubiquitin ligases, including MuRF1 and atrogin-1, which are involved in protein degradation [41]. Consequently, to further investigate these processes, the mRNA expression levels of factors related to muscle atrophy were analyzed using real-time qPCR. As shown in Fig. 3, DEX treatment led to the downregulation of MHC isoform expression, whereas the expression of genes associated with the protein degradation pathway, such as MuRF1, atrogin-1, and foxO3a, was upregulated. Conversely, the expression levels of atrogin-1 (*p* < 0.01), MuRF1, and foxO3a (*p* < 0.001) were significantly reduced following EFEL6501 treatment (Fig. 3A-3C). Additionally, treatment with EFEL6501 significantly restored the expression levels of MHC (*p* < 0.05), as well as MHC IIa, MHC IIb, and MHC IIx (*p* < 0.001), compared to the DEX group (Fig. 3D-3G). These results indicate that EFEL6501 treatment alleviates DEX-induced muscle atrophy by promoting muscle contractile protein synthesis while suppressing proteolytic gene expression.

### EFEL6501 Improves the Levels of Muscle-Related Proteins in Serum

**Fig. 4:**
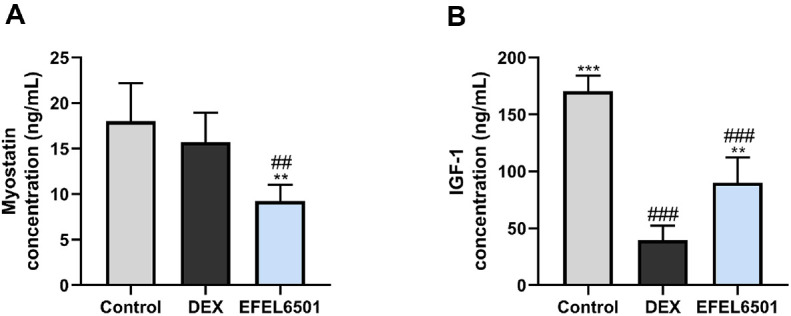
*Lacticaseibacillus paracasei* EFEL6501 attenuates the expression of muscle degradation and promotes muscle differentiation-related protein in serum. The protein levels of (**A**) myostatin and (**B**) insulin-like growth factor (IGF-1) were measured. The enzyme-linked immunosorbent assay (ELISA) analysis was performed in triplicate. Values are represented as mean ± SD (*n* = 5). **p < 0.01, ***p < 0.001 vs. DEX group; ^##^*p* < 0.01, ^###^*p* < 0.001 vs. control group. Control, treated with saline; DEX, dexamethasone (DEX)-treated with starch; EFEL6501, DEX-treated with 10^8^ CFU of *Lacticaseibacillus paracasei* EFEL6501.

Serum protein levels induced by DEX were quantified using ELISA. Numerous studies have reported that the inhibition of myostatin leads to an increase in muscle mass but a decline in muscle quality [42, 43]. Additionally, IGF-1 is a key growth factor that regulates muscle size and function by modulating both anabolic and catabolic pathways [44]. Based on these findings, the administration of EFEL6501 improved DEX-induced myostatin levels compared with the DEX group (*p* < 0.01) (Fig. 4A). In contrast, as shown in Fig. 4B, EFEL6501 significantly increased IGF-1 levels compared with the DEX group (*p* < 0.01). These findings suggest that EFEL6501 ameliorates DEX-induced muscle atrophy, thereby regulating key factors involved in muscle growth and degradation.

### EFEL6501 Increases the Cross-Sectional Area of Gastrocnemius Muscle Fibers

**Fig. 5:**
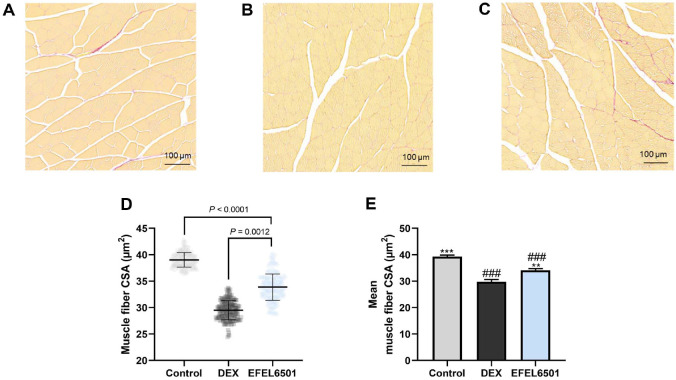
*Lacticaseibacillus paracasei* EFEL6501 recovers the cross-sectional areas of gastrocnemius muscle fibers. (**A-C**) Histological analysis of gastrocnemius muscle fibers using Sirius Red staining, with fiber cross-sectional area (CSA) (μm^2^) quantified using ZEN 3.1 software (scale bars = 100 μm). (**D**) Quantification of muscle fiber CSA (μm^2^) and (**E**) average CSA (μm^2^) calculated from 200 muscle fibers per group. Values are represented as mean ± SD (*n* = 3). **p < 0.01, ***p < 0.001 vs. DEX group; ^###^*p* < 0.001 vs. control group. Control, treated with saline; DEX, dexamethasone (DEX)-treated with starch; EFEL6501, DEX-treated with 10^8^ CFU of *Lacticaseibacillus paracasei* EFEL6501.

To assess the atrophic condition of gastrocnemius muscle induced by DEX, Sirius Red staining was performed on muscle fibers. Representative images from each group showed no clear differences in fiber size across the three groups (Fig. 5A-5C). Meanwhile, quantitative analysis of the CSA demonstrated that EFEL6501 significantly increased muscle fiber size to 34.11 μm^2^ compared with 29.79 μm^2^ in the DEX group (*p* < 0.01) (Fig. 5D and 5E). These results indicate that EFEL6501 alleviates DEX-induced muscle atrophy by restoring the CSA of gastrocnemius muscle fibers.

### EFEL6501 Enhances Intestinal Microbial Diversity by Promoting Beneficial Gut Bacteria

**Fig. 6:**
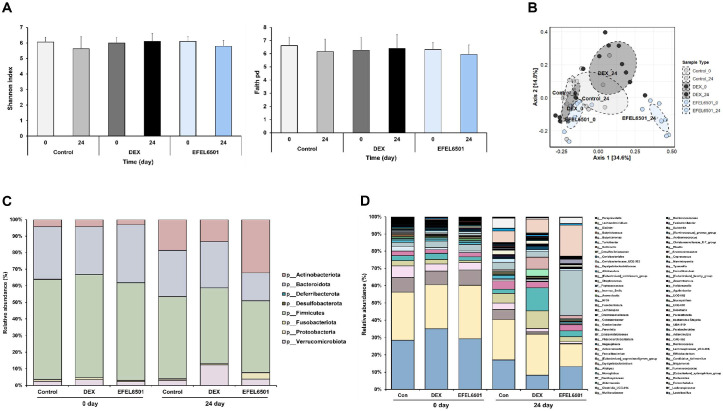
*Lacticaseibacillus paracasei* EFEL6501 influences gut microbial composition in a dexamethasone-induced muscle atrophy mouse model. Fecal samples were collected from mice on days 0 and 24 for 16S ribosomal RNA (16S rRNA) sequencing. (**A**) Alpha diversity measured using the shannon index and faith's phylogenetic diversity (Faith PD), with error bars representing standard deviation (*n* = 7). (**B**) Beta diversity was assessed using principal coordinates analysis (PCoA) plots based on Bray-Curtis distances. Relative abundance of gut microbiota at (**C**) the phylum level and (**D**) genus level. Control, treated with saline; DEX, dexamethasone (DEX)-treated with starch; EFEL6501, DEX-treated with 10^8^ CFU of *Lacticaseibacillus paracasei* EFEL6501.

**Fig. 7:**
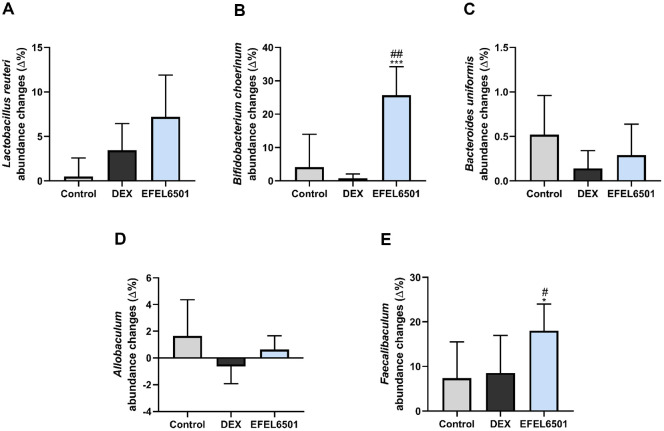
*Lacticaseibacillus paracasei* EFEL6501 modulates the relative abundance of gut microbiota at the genus and species levels in a dexamethasone-induced muscle atrophy mouse model. Changes in the relative abundance of gut microbiota at the genus or species level on days 0 and 24 are shown. (**A**) *Lactobacillus reuteri*, (**B**) *Bifidobacterium choerinum*, (**C**) *Bacteroides uniformis*, (**D**) *Allobaculum*, and (**E**) *Faecalibaculum*. Values are represented as mean ± SD (*n* = 7). *p < 0.05, ***p < 0.001 vs. DEX group; ^#^*p* < 0.05, ^##^*p* < 0.01 vs. control group. Control, treated with saline; DEX, dexamethasone (DEX)-treated with starch; EFEL6501, DEX-treated with 10^8^ CFU of *Lacticaseibacillus paracasei* EFEL6501.

To evaluate changes in gut microbiota associated with DEX-induced muscle atrophy, we assessed microbial diversity and taxonomic differences. As shown in Fig. 6A, α-diversity analysis (Shannon and Chao1) revealed no significant differences among the experimental groups. In contrast, the principal coordinate analysis (PCoA) plot in Fig. 6B demonstrated distinct clustering between the DEX and EFEL6501 groups after 24 days, suggesting that EFEL6501 influenced the gut microbiota composition altered by DEX treatment. Consistent with these findings, analysis at the phylum and genus levels indicated a notable increase in the genus *Bifidobacterium*, belonging to the phylum *Actinobacteria*, following EFEL6501 treatment (Fig. 6C and 6D). Similarly, the relative abundance of *Bifidobacterium choerinum* markedly increased in the EFEL6501 group compared with both the control (*p* < 0.01) and DEX groups (*p* < 0.001) (Fig. 7B). In addition, *Lactobacillus reuteri*, *Bacteroides uniformis*, and *Allobaculum* tended to increase in the EFEL6501 group, although these changes were not statistically significant (Fig. 7A, 7C, and 7D). Notably, *Faecalibaculum* was significantly elevated in the EFEL6501 group compared with both the control (*p* < 0.05) and DEX group (*p* < 0.05) (Fig. 7E). These results indicate that EFEL6501 treatment restored the gut microbiota composition disrupted by DEX treatment by increasing the relative abundances of beneficial bacteria (Table 2).

### EFEL6501 Alters Gut Metabolic Profiling by Modulating Bioactive Metabolite Production

The effects of EFEL6501 on changes in gut metabolites in a DEX-induced muscle atrophy model were analyzed using GC-MS. After 24 days, acetate levels markedly increased in the EFEL6501 group, whereas the DEX group showed a significant decrease (*p* < 0.01) (Table 3). This resulted in a 96% higher acetate level in the EFEL6501 group compared to the DEX group. However, the concentrations of butyrate, propionate, and formate remained unchanged after 24 days in all groups. Therefore, that EFEL6501 administration selectively increases acetate levels in the gut metabolite profile of the DEX-induced muscle atrophy model.

## Discussion

In 2019, the European Working Group on Sarcopenia in Older People (EWGSOP2) defined sarcopenia as a condition characterized by reduced muscle strength, low muscle mass or quality, and impaired physical performance [45]. Dysbiosis of gut microbiota, which is frequently observed in older adults, has been associated with chronic inflammation and anabolic resistance, both of which contribute to muscle degradation [46, 47]. Given this connection, probiotics have gained attention as a potential nutritional intervention for preventing and managing sarcopenia by restoring gut microbiota balance and enhancing muscle function [48]. In this study, the EFEL6501 strain demonstrated promising potential as a probiotic candidate for supporting muscle health by alleviating muscle atrophy through the regulation of gut microbiota.

Glucocorticoids, such as DEX, are known to induce muscle atrophy by inhibiting the PI3K–AKT signaling pathway, which subsequently activates FOXO. This activation induces the expression of muscle-specific E3 ubiquitin ligases, including MuRF1 and atrogin-1, which drive protein degradation and muscle mass loss [49, 50]. To prevent or mitigate muscle atrophy induced by high-dose glucocorticoid administration, probiotics have increasingly been recognized as potential modulators of muscle health. Probiotics exert beneficial effects not only by the viable microorganisms themselves but also by bioactive components derived from non-viable cells and cell-free extracts, which are generally termed postbiotics [51]. Among these, probiotic-derived metabolites, such as SCFAs and EPS, together with structural components including peptidoglycans and teichoic acids, represent key factors mediating host–microbe interactions [52]. Recently, several studies have suggested that these bioactive molecules can modulate muscle function by alleviating atrophy-related pathways and promoting muscle regeneration [53, 54]. In line with these findings, our study demonstrated that CS and LS of EFEL6501 treatment significantly increased the expression of myogenesis-related factors while downregulating atrogin-1 expression in DEX-treated C2C12 myotubes (Fig. 1), indicating a potential protective role against glucocorticoid-induced muscle degradation. Similarly, in the DEX-induced mouse model, EFEL6501 administration restored the expression levels of muscle atrophy-related factors, which had been elevated following DEX treatment, to normal levels. Furthermore, the expression of the muscle contractile protein MHC was significantly upregulated, suggesting improved muscle function (Fig. 3). These molecular changes were accompanied by a significant recovery in both muscle strength and muscle thickness following EFEL6501 treatment (Fig. 2F and 2G), further supporting its potential efficacy in preserving muscle integrity under glucocorticoid-induced atrophic conditions.

Additionally, muscle growth is regulated by various stimuli, including insulin, IGF-1, and amino acid signaling, which ultimately activate the PI3K–AKT–mammalian target of rapamycin (mTOR) axis. This signaling pathway plays a crucial role in maintaining cellular homeostasis by inhibiting protein degradation and shifting muscle protein metabolism toward protein synthesis [55]. DEX, commonly used to induce muscle atrophy, suppresses IGF-1 and insulin-like growth factor binding protein 3 (IGFBP3), thereby inhibiting PI3K–AKT–mTOR signaling [56]. Moreover, DEX treatment has been shown to upregulate the myostatin/ transforming growth factor-β (TGF-β) signaling pathway, leading to Smad2/3 activation. This, in turn, inhibits Akt function via FOXO transcription factors, thereby promoting the expression of atrogin-1 and MuRF1, key regulators of muscle protein degradation [57]. Consistent with these mechanisms, our study demonstrated that EFEL6501 treatment significantly reduced serum myostatin levels while restoring IGF-1 levels in mice (Fig. 4). These findings suggest that EFEL6501 counteract two opposing molecular pathways involved in muscle homeostasis, reactivating the IGF-1/PI3K/AKT/mTOR axis to promote protein synthesis while suppressing the myostatin/TGFβ–Smad2/3–FOXO pathway to inhibit protein degradation. Similarly, whey protein fermented with *L. gasseri* IM13 was found to enhance the mRNA expression levels of IGF-1 and IGFBP3 [22].

Recent research has underscored the gut microbiota as a potential target for modulating the biological processes underlying aging. In parallel, the gut-muscle axis has gained increasing attention as a key factor in understanding the complex interplay between gut microbiota and skeletal muscle function [58]. In this study, EFEL6501 administration led to a notable increase in SCFA-producing bacterial genera, including *Lactobacillus*, *Bifidobacterium*, *Allobaculum*, and *Faecalibaculum*, suggesting their potential role in mitigating muscle atrophy (Table 2 and Fig. 7). These findings align with previous studies reporting a significant reduction in *Bifidobacterium pseudocatenulatum*, *Bifidobacterium longum*, and *Bifidobacterium adolescentis*, which are beneficial gut bacteria, in individuals diagnosed with sarcopenia. In addition, a marked depletion of *Bi. adolescentis* in sarcopenic individuals has been linked to declines in muscle mass and function [59]. Furthermore, *Allobaculum*, which is abundantly found in rodents, is an active glucose metabolizer known to produce lactate and butyrate [60, 61], and its relative abundance has been significantly associated with aging [62]. Additionally, although *Faecalibaculum* remains relatively understudied, it has been found to inhabit the ileal mucus layer of rodents and contribute to tumor suppression in mice through the production of SCFAs in the colon [63]. These results indicate that EFEL6501 modulates gut microbiota composition by promoting the proliferation of beneficial bacteria, which may contribute to alleviating muscle atrophy.

Beneficial gut bacteria primarily produce SCFAs as key metabolites, among which acetate, propionate, and butyrate are the most abundant [64]. The depletion of these beneficial microbes and their metabolites can lead to gut barrier dysfunction, allowing inflammatory toxic substances to translocate into the systemic circulation and contribute to muscle damage [65]. Among SCFAs, acetate has been reported to play a dual role in skeletal muscle by promoting both lipolysis and glycogen synthesis [66, 67]. Notably, lower acetate levels have been observed in individuals with sarcopenia, and acetate supplementation has been shown to preserve grip strength and muscle fiber CSA, thereby mitigating muscle weakness and atrophy in antibiotic-treated and low-fiber diet models [68, 69]. In line with these findings, our results showed a significant increase in acetate levels in the EFEL6501-treated group compared with the DEX group after 24 days (Table 3). This effect was accompanied by an enrichment of acetate-producing bacteria such as *Faecalibaculum*, *Lactobacillus*, and *Bifidobacterium*, suggesting that EFEL6501 may ameliorate muscle atrophy through targeted modulation of gut microbiota and enhanced acetate production.

Despite the significance of our findings, this study has certain limitations that should be considered. The *in vitro* experiments used LS and CS containing bioactive compounds produced by the EFEL6501 strain. Therefore, further studies are required to elucidate how specific bioactive compounds within these substances influence muscle physiology. Identifying the specific bioactive components responsible for the observed *in vitro* effects remains a challenge. Further studies could help determine which components of EFEL6501 contribute to mitigating muscle atrophy. Subsequently, the identified bioactive compounds should be isolated and tested in *in vivo* models to clarify the role of EFEL6501 in muscle atrophy prevention and treatment.

## Conclusion

In this study, EFEL6501 demonstrated anti-atrophic effects in both DEX-induced C2C12 myotubes and mouse models. Treatment with EFEL6501 effectively mitigated DEX-induced muscle atrophy and improved muscle performance. This effect was particularly associated with the suppression of muscle protein degradation, resulting in enhanced strength, myogenic differentiation, and increased muscle fiber CSA. In addition, EFEL6501 administration contributed to the restoration of gut microbiota composition and the normalization of microbial metabolite profiles altered by DEX treatment. Taken together, these results support the potential of EFEL6501 as a promising probiotic candidate for muscle health.

## Figures and Tables

**Table 1:** Primer sequences used for real-time qPCR.

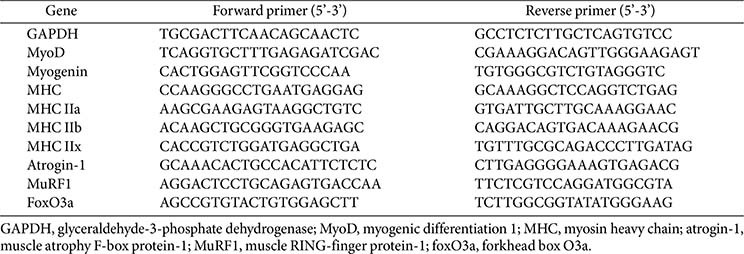

**Table 2:** Changes in the relative abundance of gut bacterial genera and species in a dexamethasone-induced muscle atrophy mouse model.

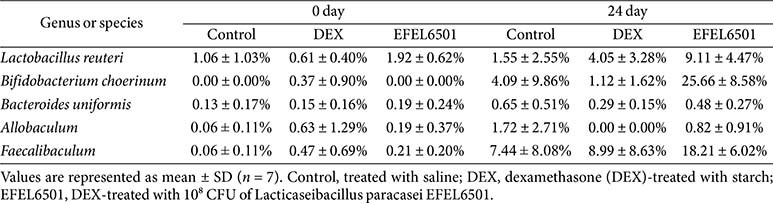

**Table 3:** Changes in short-chain fatty acid concentrations induced by *Lacticaseibacillus paracasei* EFEL6501.